# Impending Mental Health Issues During Coronavirus Disease 2019 – Time for Personalized Nutrition Based on the Gut Microbiota to Tide Over the Crisis?

**DOI:** 10.3389/fnins.2021.831193

**Published:** 2022-01-17

**Authors:** Debojyoti Dhar

**Affiliations:** Leucine Rich Bio Pvt. Ltd., Bengaluru, India

**Keywords:** COVID-19, mental health, gut microbiome, nutrition, precision medicine

## Abstract

Coronavirus disease 2019 (COVID-19) is a major pandemic facing the world today caused by SARS-CoV-2 which has implications on our mental health as well. The uncertain future, fear of job loss, lockdown and negative news all around have taken a heavy toll on the mental health of individuals from across the world. Stress and anxiety can affect the COVID-19 patients even more. Recent study suggests COVID-19 infection may lead to post-traumatic stress disorder (PTSD). Certain prebiotics and probiotics have been shown to have anxiolytic effect through gut microbiota modulation. Incidentally, preliminary report also suggests a differential microbial profile in COVID-19 patients as compared to healthy individuals. Gut microbiota’s role in anxiety and depression is well studied. The importance of the “gut-brain” axis has been implicated in overall mental health. It is known that diet, environmental factors and genetics play an important role in shaping gut microbiota. Trials may be initiated to study if personalized diet and supplementation based on individual’s gut microbiome profile may improve the general mental well-being of people prone to anxiety during this pandemic. Also, COVID-19 patients may be provided personalized nutritional therapy based on their gut microbiota profile to see if PTSD and anxiety symptoms can be alleviated.

## Introduction

**FIGURE 1:**
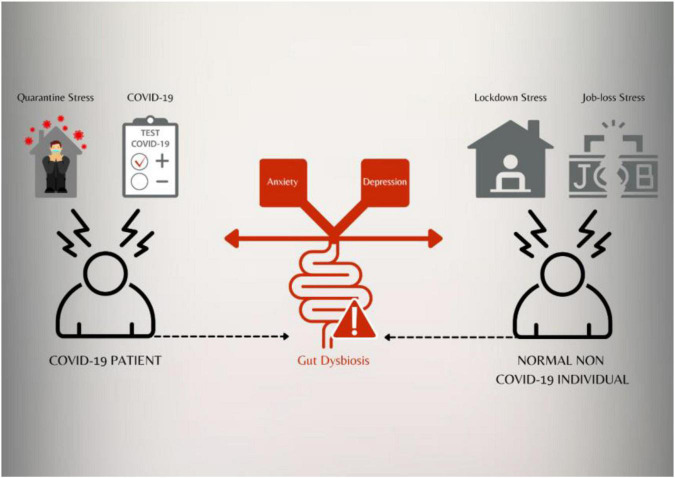
Coronavirus disease 2019 itself and various stressors may impact the gut microbiota which can lead to gut dysbiosis. Gut dysbiosis may be implicated in anxiety and depression both in COVID-19 patients and healthy individuals exposed to various stressors.

Coronavirus disease 2019 (COVID-19) is a raging pandemic causing a widespread disruption of normal life. Although, the first case was reported in the Hubei province of China in late 2019 yet it has spread to many countries in the world (Wang et al., 2020). Apart from the clinical symptoms that this disease manifest, it has now been reported that COVID-19 infection might lead to post-traumatic stress disorder (PTSD; Rogers et al., 2020). Even the healthcare workers who are at the forefront of managing this disease have reported an increased prevalence of anxiety, depression, and insomnia (Pappa et al., 2020). Not only COVID-19 is affecting the mental health of patients and the frontline healthcare workers, this pandemic is taking a heavy mental health toll on people from across the world (Figure 1). The uncertainties of the future, the job losses, the extended lockdown and the overall negative environment all around is causing a massive jump in the number of anxiety and depression cases in the world (Taquet et al., 2021). In a recent research study in India, it was found that the adolescents who were quarantined and children experienced more psychological distress than the non-quarantined children and adolescents (Saurabh and Ranjan, 2020). Another international study covering 9565 people across 78 countries found people affected with low or moderate mental health during COVID-19, suggesting a major impact of the pandemic on mental well-being of majority of people across the world (Gloster et al., 2020).

Stress can trigger development of anxiety and depressive-like behavior (Yang et al., 2015). The current pandemic situation with lockdowns, quarantine processes, and misinformation can all add to the stress levels. The prevailing therapeutic options for treating mood disorders like anxiety and depression include the tricyclic antidepressants (TCAs), selective serotonin reuptake inhibitors (SSRIs), serotonin-norepinephrine reuptake inhibitors (SNRIs), and the classical benzodiazepines (Busato et al., 2006). However, all of these drugs take a few days to weeks to show their affect and may not show positive effects in many and in few cases can cause adverse effects causing compliance issues (Balikci et al., 2014). Hence, it is imperative that alternatives that can be used alone or in conjunction with the current therapies should be studied and implemented. Herbal nootropics like Ashwagandha or *Withania somnifera*, certain prebiotics and probiotics have been shown to have anxiolytic effect *via* modulation of the gut microbiome (Peterson et al., 2019). Prebiotics are defined as non-digestible dietary fibers that can confer benefit to the host gut health by selectively stimulating growth of indigenous health promoting bacteria (Simpson and Campbell, 2015). Similarly, probiotics are defined as live non-pathogenic microorganisms like bacteria and yeast that exhibit beneficial health effects to the host when consumed in adequate amount (Cukrowska et al., 2009). Alterations in bacterial community metabolism as a consequence of medicinal herb-driven prebiotic may contribute to therapeutic efficacy (Peterson et al., 2019). Differential gut microbiota profile has been found in patients with general anxiety disorder (GAD) and major depressive disorder (MDD) (Jiang et al., 2018; Winter et al., 2018). Although, studies in animal models suggest a causal role of the gut microbiota in accentuating such mental conditions, more research and studies are needed to completely understand the mechanisms. COVID-19, with its impending effect on the psychological health of both the patients and millions of individuals worldwide, provides the necessary impetus to the scientific and the medical community to delve deeper into the role of the gut microbiota in depression and anxiety. This article is an effort to drive in the fact that gut microbiota-based nutritional supplementation solutions need to be looked into in greater depth as the current crisis has left open a deep gap in the health infrastructure especially dealing with mental health issues. In the subsequent paragraphs, I would try to highlight the current understanding with respect to the role of the gut microbiota in mental health conditions such as anxiety and depression and also the possibility of personalized nutrition solutions based on the gut microbiota profile of an individual to address the issues of mental well-being in COVID-19 times. This approach may be suitable for both the patients who are affected by the disease (COVID-19) and the general population who are vulnerable to anxiety and depression especially due to the conditions in this current pandemic.

## Brain, Gut, and the Microbiome – Current Understanding

There is a complex communication between the microorganisms in the gastrointestinal tract and the peripheral and the central nervous system (CNS). The gut consists of the enteric nervous system (ENS) that can act independently of the brain and the spinal cord (Furness, 2012). The gut microbiota are the major component of the “gut-brain axis” that includes various pathways that enable communication between the intestine or the gut and the CNS (Misiak et al., 2020). The human intestinal microbiota consists of 10^14^ microorganisms including bacteria, fungi, viruses, and archaea (Gill et al., 2006). The intestinal bacteria in healthy individuals is dominated by four phyla belonging to Actinobacteria, Firmicutes, Proteobacteria, and Bacteroidetes (Villanueva-Millán et al., 2015). The gut microbiota of adult humans is dominated by members of the Firmicutes and the Bacteroidetes phyla (Hall et al., 2017). The intestinal microbiota plays a key role in health and wellness through its protective, and metabolic actions (Cani, 2018).

“Gut dysbiosis” or alterations of gut microbiota have been shown to be associated with various diseases and disorders like inflammatory bowel disease (IBD; Khan et al., 2019), type 2 diabetes (Gurung et al., 2020), cardiovascular disease (Tang et al., 2017), and even mental disorders like depression and anxiety (Zalar et al., 2018; Peirce and Alviña, 2019). Incidentally, preliminary report also suggests a differential microbial profile in COVID-19 patients as compared to healthy individuals (Gu et al., 2020; Zuo et al., 2020). Hence, it is important to study the effect of such dysbiosis in mental health conditions of COVID-19 patients. This is mainly because gut dysbiosis has been implicated in depression and anxiety (Clapp et al., 2017). More so, when COVID-19 patients and healthcare providers have shown bouts of depression, anxiety, and stress (Pfefferbaum and North, 2020; Rogers et al., 2020).

The bidirectional “gut-brain axis” communication is known to involve neural (Vagus nerve and ENS), immune (cytokine), and endocrine [cortisol and hypothalamic–pituitary–adrenal (HPA) axis] pathways (Mörkl et al., 2020). Studies in germ-free (GF) mice have shown that gut microbiota are essential for development of neuronal circuits, anxiety behavior, and social responses (Das et al., 2017).

### The Hypothalamic–Pituitary–Adrenal Axis – Modulation by the Gut Microbiota

The intestinal microbiota may determine the stress responsivity by modulating the HPA axis (Peirce and Alviña, 2019). The HPA axis gets initiated in the hypothalamus region of the brain by the synthesis of corticotrophin-releasing hormone (CRH). CRH then stimulates the production of the adrenocorticotrophic hormone (ACTH) in the pituitary gland which then leads to release of the glucocorticoids from the adrenal cortex. Higher stress levels are known triggers of anxiety and depression (Rea et al., 2016). Research and studies on GF mice have shed a lot of light on the role of the gut microbiota in influencing the HPA axis. A pivotal study reported that GF mice have an overreactive HPA axis which leads to increased concentrations of ACTH and corticosterone following a stressful stimulus (Sudo et al., 2004). Other studies have shown a differential gene expression pattern in GF mice as compared to the control mice especially in the regions of hippocampus and cortex, which most likely influence the difference in HPA axis activity (Heijtz et al., 2011; Bellavance and Rivest, 2014). It has been shown that various stressors or stressful stimuli may impact the abundance of *Lactobacilli*, *Bacteroides*, and *Clostridium* in animal models affecting barrier integrity as well (Misiak et al., 2020). Studies have shown that probiotics based on *Bifidobacterium* and *Lactobacillus* species restore stress-induced HPA axis dysfunction and improve depression- and anxiety-like symptoms (Eutamene et al., 2007; Desbonnet et al., 2010). The intestinal microbiota can influence the HPA axis by increase in levels of cytokines, release of LPS and peptidoglycan (cell wall components of the bacteria) and by short chain fatty acid (SCFA) production (Misiak et al., 2020). Interestingly, it has been found that abnormal activation of the HPA axis impacts microbial colonization (Pellissier and Bonaz, 2017). Also, evidence from animal model studies points to the fact that stress-related HPA axis response may increase the intestinal permeability (Vicario et al., 2012). Although, less is known about a cross-talk between the HPA axis and the gut microbiota in major depression, a previous study showed transplantation of the gut microbiota from patients with depression to GF mice was associated with the development of anxiety- and depression-like phenotype. This was accompanied by a down-regulation of the Stat5a gene in the hippocampus region of the brain (Luo et al., 2018). Interestingly, the Stat5a gene is known to regulate the HPA axis response. Furthermore, a murine model of depression has been reported to overproduce CRH indicating a hyper active HPA axis (Park et al., 2013). Although many preclinical studies have included GF and antibiotic treated mice, it is suggested that such treatment or microbiota free environment might lead to certain changes at the cellular/organ level and hence there are certain caveats that need to be kept in mind (Kennedy et al., 2018). Taken together, the evidence suggests the role of the gut microbiota in influencing the HPA axis that might lead to development of the “anxiety-depression” state. Conversely, stress mediated HPA activation can also affect the intestinal microbiota which again can lead to dysregulation of HPA axis and other pathways leading to the above disease conditions. Considering the various stressors in the current COVID-19 pandemic, it is tempting to speculate that this might be impacting the HPA axis and hence the role of the gut microbiota in such a scenario becomes important to study.

### Inflammation and the Immune System – Possible Role of the Gut Microbiota

The interactions of the host with the microbes are complex and is also bidirectional. The intestinal microbiota are thought to regulate the development and function of the innate and adaptive immune system (Negi et al., 2019a). Intestinal micro-organisms secrete antimicrobial peptides such as bacteriocins, compete for the nutrients and the habitat site thereby aiding in the state of homeostasis (Moens and Veldhoen, 2012). GF mice studies have provided important insights into the role of the resident microbiota in host immune system development. For example, GF mice have been shown to have an under-developed mucosal immune system. Also, GF mice have been found to have lesser number of regulatory T cells (Tregs) with reduced anti-inflammatory activities (Strauch et al., 2005). In the CNS, the microglia are a kind of immune cells that post activation can release lot of cytokines and chemokines, regulate neurotransmitters and can undergo morphological changes (Rea et al., 2016). Stress induces glucocorticoid (cortisol in humans) secretion and it is interesting to note that glucocorticoid receptors are expressed abundantly on microglia throughout the brain (Sierra et al., 2008). Cytokines release post microglia activation have been found to have a role in behavioral phenotype in stress models (Kreisel et al., 2014). The gut microbiota and immune homeostasis are intertwined and this relationship is also a domain of great interest and research. It is suggested that decrease in gut diversity and change in the normal gut microbiota profile may alter normal immune function (Dhar and Mohanty, 2020). Also, signals derived from the intestinal microorganisms can tune the immune cells for pro and anti-inflammatory responses thereby affecting the susceptibility to various diseases (Negi et al., 2019b). It is documented that the immune gut homeostasis is controlled by the intestinal microbiota by the fine tuning of the regulatory balance of pro-inflammatory responses such as Th17 versus anti-inflammatory Tregs (Round and Mazmanian, 2010). The role of inflammation in depression is well documented in various studies. In fact, genetic variants in various immune related genes have been implicated in depression (Barnes et al., 2017). Studies have also reported correlation between higher pro-inflammatory cytokines and depression in humans (Lamers et al., 2013). It is suggested that breach in gut barrier integrity may lead to the translocation of bacteria and bacterial antigens (such as lipopolysaccharides) into the blood stream causing chronic low-grade inflammation (Mörkl et al., 2020). Stress increases intestinal permeability in several animal models (Yu et al., 2013). This may lead to endotoxins and other harmful bacteria to seep into the circulation causing an immune reaction and inflammation. Many animal studies have shown that administration of endotoxins peripherally causes global expression of pro-inflammatory cytokines in the brain (Peirce and Alviña, 2019). The other mechanisms through which peripheral inflammation spread to the brain and cause neuro-inflammation can be by sending inflammatory signals to the brain by afferent nerves, activated immune cells migrating to the brain and cytokines crossing the blood brain barrier (Peirce and Alviña, 2019).

### The Vagus Nerve and the Gut Microbiota – Is There a Connection?

The Vagus nerve innervates a large proportion of the body’s digestive tract and is known to be responsive to a number of endogenous chemicals emanating in the digestive tract (Bonaz et al., 2018). The Vagus nerve is known to relay signals from the brain to the viscera. Interestingly, approximately 80% of Vagus nerve fibers are afferent, relaying sensory information from the viscera, including the digestive tract, to the brain to maintain homeostasis (Winter et al., 2018). Microbiota secretions can activate Vagal afferents which then signal to the hypothalamic regions of the brain (Forsythe et al., 2014). Evidence suggests that for probiotics to mediate beneficial effects in anxiety and depression, intact Vagus nerve is required. Example, a study revealed that mice with inflammation in the intestine that normally exhibited anxiety-like behavior showed less anxiety symptoms when treated with *Bifidobacterium longum*; however, this anxiolytic effect was not observed in mice in which the Vagus nerve was severed (Bercik et al., 2011). Similar observation was found with the probiotic *Lactobacillus rhamnosus* in mice (Bravo et al., 2011). Incidentally, the anxiolytic property of the probiotic *B. longum* has also been observed in humans (Allen et al., 2016). It is also possible that pathogenic microbes might modulate Vagal afferents causing subsequent pathologic changes in the CNS, which may then lead to anxiety/depression like diseases (Winter et al., 2018). Similarly, there are multiple studies conducted in mice which suggest the role of the vagus nerve in depressive behavior (Pu et al., 2021; Wang et al., 2021). Taken together, the evidences point out to the possible role the gut microbiota may play in modulating the Vagus nerve thereby influencing the mental diseases like depression and anxiety.

## Diet and Probiotics/Prebiotics – Need a Personalized Approach

Intestinal microbiota is considered malleable and can be modulated by diet, medication, lifestyle, environment, etc. The type of food that we eat is known to play an important role in shaping the composition of the gut microbiota. Diet is found to influence the specific compositional patterns of the gut microbiota based on the nutritional components of the food like, e.g., the different composition of the microbiota with animal fat and protein-based diets versus vegetable-based diets (De Filippis et al., 2016). Systemic stress and chronic inflammation can also differentially affect the gut microbiota thereby proving that environmental factors along with diet can modulate the composition of the gut microbiome (Earley et al., 2015). Dietary fats, particularly trans, and saturated fats, are known to transiently increase intestinal inflammation (Okada et al., 2013). This in turn alters gut microbial population by increasing pathogenic and decreasing commensal taxa (Das et al., 2017). High fat, low fiber diet is also known to decrease gut microbiome diversity (Simpson and Campbell, 2015). Therefore, it is important to have a balanced diet rich in diverse and plant-based products which would likely lead to a more diverse, balanced and resilient microbiome composition in the gut. This would eventually have an impact on the mental health of the individual as well. In the context of COVID-19, it was found that specific formulation of probiotics containing various strains of *Streptococcus thermophilus* DSM 32345, *L*actobacillus *acidophilus* DSM 32241, *Lactobacillus helvetics* DSM 32242, *Lactobacillus paracasei* DSM 32243, *Lactobacillus plantarum* DSM 32244, *Lactobacillus brevis* DSM 27961, *Bifidobacterium lactis* DSM 32246, *B. lactis* DSM 32247 reduced the risk of progression to severe COVID-19 in patients treated with the special probiotic formulation as compared to the patients not given the oral bacteriotherapy (d’Ettorre et al., 2020). Although the psychological parameters were not evaluated in this study yet this study provided the proof of concept of the role of the probiotics in improving the outcome in COVID-19 patients. Few more studies are being conducted to find the effect of nutritional supplements known to modulate the gut microbiota in treating COVID-19 (Table 1). In COVID-19 context therefore, one of the ways by which stressors can be neutralized is by following a diet and supplement intake based on the individual’s gut microflora.

**TABLE 1:** Select studies with nutritional supplements that modulate the gut microbiota in treating COVID-19.

Study	Evaluation	Clinical trial identifier
Modulation of gut microbiota to enhance health and immunity of vulnerable individuals during COVID-19 pandemic	Double-blinded, randomized, active-placebo controlled study for evaluation of the efficacy of modulating the gut microbiota with a specific probiotic composition (3 bifidobacteria, 10 billion cfu per sachet) in COVID-19 patients with comorbidity like type 2 diabetes and elderly	NCT04884776
An exploratory, open label, clinical study to evaluate the physiologic effects of KB109 in adult patients with mild-to-moderate COVID-19 on gut microbiota structure and function in the outpatient setting	Evaluation of the modulatory effect of the glycan KB109 in mild to moderate COVID-19 patients	(Haran et al., 2021); NCT04486482
Modulation of gut microbiota with NBT-NM108 as an early treatment for suspected or confirmed symptomatic COVID-19 patients	Open labeled, randomized, and controlled clinical trial for evaluation of a novel botanical based fixed combination drug – NBT-NM 108 in modulating the gut microbiota and treat early stage COVID-19 patients	NCT04540406
The intestinal microbiota as a therapeutic target in hospitalized patients with COVID-19 infection	Prospective case-control pilot study for evaluation of a specific probiotic (1 billion cfu) mix to improve outcome in COVID-19 patients	NCT04390477
Multicentric study to assess the effect of consumption of *Lactobacillus coryniformis* K8 on healthcare personnel exposed to COVID-19	Preventive study to evaluate the effect of consumption of the probiotic *Lactobacillus coryniformis* K8 (3 billion cfu) in incidence and severity of COVID-19 in health workers exposed to the virus	NCT04366180
Evaluation of the efficacy of probiotics to reduce the duration and symptoms of COVID-19 (PROVID-19 study): a randomized, double-blind, controlled trial	Randomized controlled trial to evaluate the efficacy of probiotics (2 strains 10 × 10^9^ UFC) to reduce the duration and symptoms of COVID-19	NCT04621071
Efficacy of probiotics in treatment of hospitalized patients with novel coronavirus infection	A randomized controlled open-label study for evaluation of probiotics *L. rhamnosus* PDV1705 (1 billion cfu), *Bifidobacterium bifidum* PDV 0903 (1 billion cfu), *B. longum* PDV 1911 (1 billion cfu), *B. longum* PDV 2301 (1 billion cfu) in treatment of patients hospitalized with COVID-19	NCT04854941

The term “Psychobiotics” now define all microbiota targeting interventions including prebiotics and probiotics that can influence bacteria-brain relationship (Sarkar et al., 2016). Many preclinical studies have investigated the role of prebiotics and probiotics in mental health. A study has reported that a combination of prebiotics, fructooligosachharide (FOS) and galactooligosaccharide (GOS) attenuated anxiety related behavior in mice (Rea et al., 2016). Similarly, administration of GOS and polydextrose to rats prevented anxiety and depression like behavior (Mika et al., 2017). Many preclinical studies have illustrated positive role of probiotics in anxiety and depression. Colonizing GF mice with *Bifidobacterium infantis* stabilizes their overreactive HPA axis in response to restraint stress and returns their stress hormone levels to normal as observed in control mice (Sudo et al., 2004). Resilience to stress is another aspect where probiotics have been shown to have a positive effect in preclinical studies. It was found that *Bifidobacterium* might play a role in resilience in mice subjected to chronic social defeat stress (Yang et al., 2017). Probiotics administration has been shown to improve the intestinal integrity thereby decreasing its permeability and reducing endotoxemia. For example, mice undergoing water avoidance stress display increased intestinal permeability however treating them with *L*actobacillus *farciminis* improved gut barrier integrity and conferred epithelial and mucosal barrier strengthening (Da Silva et al., 2014). Such effects of probiotics in gut barrier integrity strengthening has been observed in humans as well. For instance, a mix of *L. rhamnosus* and *Lactobacillus reuteri* was found to reduce small intestinal permeability in children with eczema (Rosenfeldt et al., 2004). Thus, certain probiotics decrease intestinal epithelial permeability and thereby lowering the risk of endotoxemia and uncontrolled inflammation. This may have positive effects on anxiety and depression. Many probiotics have also found to have positive effects on neuroinflammation. *L. farciminis* administration to mice suppressed stress-induced neuroinflammation during partial restraint stress (Ait-Belgnaoui et al., 2014). Lot of probiotics such as *B*ifidobacterium *breve*, *Lactobacillus helveticus* NS8, *L. rhamnosus*, and *B. longum* have shown anxiolytic effects in preclinical models (Peirce and Alviña, 2019). Similar benefit of probiotics has been observed in many human studies. For example, *L. helveticus* and *B. longum* probiotic mix given to healthy human volunteers for 30 days reduced psychological distress in comparison to a control group (Messaoudi et al., 2011). Significant reduction in depression scores were found in one study where the patients were administered *B. longum* (Pinto-Sanchez et al., 2017).

**FIGURE 2:**
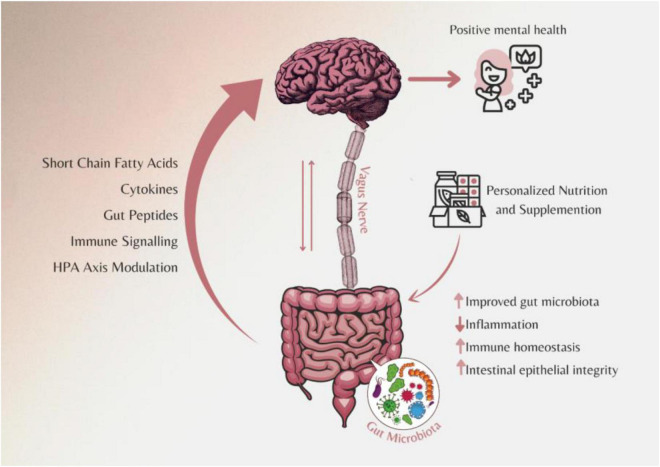
Personalized nutritional strategies including diet, prebiotics, and probiotics combination based on the individual’s gut microbiome can improve mental health conditions.

Although many studies with probiotics have shown promising results in clinical trials, still some discrepancy has been observed which might be due to different strains being used. For an effective treatment with such “psychobiotics,” it is also important to combine this with a balanced diet that would provide adequate micro and macro nutrients along with fibers. Hence, personalized nutrition with a mix of probiotics, prebiotics, and diet based on the individual’s gut microbiota may be more effective in dealing with such conditions (Figure 2).

## Conclusion and Future Perspective

Coronavirus disease 2019 has impacted everyone in the world. The world is grappling with not only the infectious nature of the disease but there is an even bigger danger looming in the background and that is the impending mental health crisis. Various meta-analysis of COVID-19 patients, frontline healthcare workers, etc., have pointed to the fact that psychological ailments like anxiety, depression, and PTSD affect them and this poses a challenge for the healthcare community (Pappa et al., 2020; Rogers et al., 2020). This along with the fact that every individual who is not infected by SARS-Cov2 virus in this planet is also affected mentally indirectly by this disease, shows the importance of tackling this crisis. Relying on the current therapies, although effective for some and with many side effects, may not be the right approach. COVID-19 has given an opportunity to the scientific and medical community to address the mental health domain by utilizing and improving on the knowledge of the gut microbiome that might provide newer strategies to counter such ailments. As evidenced, stress may lead to intestinal dysbiosis and increased gut permeability. This can lead to peripheral inflammation that can lead to neuronal inflammation in the brain. Empirical data both preclinical and clinical, suggest important role, gut microbiota might play in the mental well-being. However, there are few challenges that also need to be addressed. More studies to delineate the causal role of the microbiota in mental health need to be performed. Secondly, the role of fungi, phages should also be looked into, as much of the focus has been on bacteria. There have been few conflicting results in human trials with respect to probiotics use in alleviating depression and anxiety (Mörkl et al., 2020). This may be due to different strain of the probiotics being used in the study. Also, it is a possibility that probiotics might not work in the same way in all individuals. Host genetics, diet and colonization potential of the probiotics may also play a significant role. Hence one size might not fit all with respect to specific probiotics and prebiotics in countering anxiety and depression. Additional bigger trials in diverse population are needed to define efficacy, treatment duration, adverse effects, and dosage. Future trials might also include some aspects of genotyping to probe the effect of certain genes in probiotic colonization and their efficacy. In the current COVID-19 context, trials with personalized nutrition and supplements based on individual’s gut microflora may be initiated to check if that can improve the mental well-being of the patients both during and post recovery. The fact that there is a high burden of depression symptoms in adults especially with lower income category of society during COVID-19 pandemic suggests an overhaul of the ways by which this disease needs to be treated (Ettman et al., 2020). Overall, personalized gut microbiome based nutritional strategies, if adopted by people affected by stress and anxiety due to the prevailing environment of COVID-19 and COVID-19 patients themselves, can improve the mental well-being and might act as an alternate mode to assist the mental healthcare infrastructure which is so inadequate in developing countries.

## Data Availability Statement

The original contributions presented in the study are included in the article/supplementary material, further inquiries can be directed to the corresponding author.

## Author Contributions

The author confirms being the sole contributor of this work and has approved it for publication.

## Conflict of Interest

DD is the Director of Leucine Rich Bio (LRB), which is South Asia’s first microbiome company.

## Publisher’s Note

All claims expressed in this article are solely those of the authors and do not necessarily represent those of their affiliated organizations, or those of the publisher, the editors and the reviewers. Any product that may be evaluated in this article, or claim that may be made by its manufacturer, is not guaranteed or endorsed by the publisher.
